# The Global Physical Literacy Action Framework: Process and Outcomes

**DOI:** 10.1007/s40279-026-02485-6

**Published:** 2026-08-08

**Authors:** Johannes Carl, Lisa M. Barnett, Alethea Jerebine, Emiliano Mazzoli, Alexandre Mouton, Jaime Barratt, Peter Elsborg, Nigel Green, Marlen Niederberger, Raymond Kim-Wai Sum

**Affiliations:** 1 School of Health and Social Development, Deakin University Institute for Physical Activity and Nutrition (IPAN), Deakin University, Geelong, VIC Australia; 2 Department Health and Sport Sciences, Technical University of Munich, TUM School of Medicine and Health, Munich, Germany; 3 RUCHE – Research Unit for a life-Course perspective on Health & Education, Department of Physical Activity and Rehabilitation Sciences, University of Liège, Liège, Belgium; 4 Department of Educational Studies, Faculty of Education, Brock University, St. Catharines, Ontario, Canada; 5 Department of Epidemiology, Center for Clinical Research and Prevention, Copenhagen University Hospital – Bispebjerg and Frederiksberg, Frederiksberg, Denmark; 6 International Physical Literacy Association, Wigan, England; 7 Department Methods in Health Promotion and Prevention, Institute for Health Sciences, University of Education Schwäbisch Gmünd, Schwäbisch Gmünd, Germany; 8 Department of Sports Science and Physical Education, Faculty of Education, Chinese University of Hong Kong, Shatin, Hong Kong, China

## Abstract

**Background:**

Physical literacy integrates physical, affective, cognitive and social elements into a holistic framework for physical activity. Despite the increasing recognition of physical literacy across many sectors, the entire field lacks a prospective roadmap for research, practice and policy.

**Objective:**

Seeking to determine the content of a ‘Global Physical Literacy (GloPL) Action Framework,’ this study aimed to define goals, principles, actions and pathways that can advance the physical literacy field worldwide.

**Methods:**

A group Delphi study (based on published protocol) engaged 59 physical literacy-focused academics from 46 countries, 7 representatives of literature gaps, and 24 individuals representing societies/organisations. The representatives independently generated ideas for action through online surveys (Qualtrics). The ideas underwent reflexive thematic analysis (NVivo), informing the subsequent discussions in six online meetings. All decisions were supported by formal voting with pre-defined agreement thresholds for two rounds of voting.

**Results:**

Analysis resulted in 849 initial codes grouped into five themes: advocacy, practice, education, assessment and research. Members discussed 15 central topics, of which six reached agreement in the first voting round and nine in the second voting round. Key recommendations included: establishing a core set of physical literacy elements/principles (advocacy); generating more resources/programmes on physical literacy (practice); aligning physical education curricula with physical literacy principles (education); assessing physical literacy at the population level (assessment); and emphasising implementation science (research).

**Conclusions:**

The comprehensive consensus findings will inform the pillars of the first GloPL Action Framework. Its release marks the beginning of a dynamic process intended to guide coordinated actions on physical literacy in education, public health, sport, healthcare/promotion, rehabilitation, active recreation and community services/development.

**Supplementary Information:**

The online version contains supplementary material available at https://doi.org/10.1007/s40279-026-02485-6.

## Key Points

**Table:** 

Although the integrative concept of physical literacy has gained increasing attention worldwide, a global roadmap is essential for the future of the field.	
Based on a group Delphi methodology with different representation groups, key actions are proposed for ‘advocacy’, ‘practice’, ‘education’, ‘assessment’, and ‘research’.	
The results will directly inform the development of the first Global Physical Literacy (GloPL) Action Framework, emphasising qualitative individual experiences for promoting physical activity across diverse sectors.	

## Introduction

### Physical Activity and Physical Literacy (PL)

The fields of physical activity, exercise and sport share the common goal of fostering sustained engagement in physically active lifestyles. However, populations worldwide are insufficiently active, questioning the impact of established health promotion strategies and practices [1–3]. In recent years, physical literacy (PL) has garnered significant attention for promoting lifelong engagement in physical activity [4–6]. This multidimensional concept adopts a person-centred view on active lifestyles by encompassing physical, affective, cognitive, and sometimes social or spiritual aspects of an individual’s relationship with physical activity [7–9]. Among the different conceptualisations, the International Physical Literacy Association understands PL as “the motivation, confidence, physical competence, knowledge and understanding to value and take responsibility for engagement in physical activities for life” [10]. The Australian Physical Literacy Framework defines PL as the “holistic learning that occurs through movement and physical activity and integrates physical, psychological, social and cognitive capabilities” [11]. A Chinese consensus has specified PL as the “integration of physical, perceptual, cognitive, psychological, and behavioural capabilities, echoing the need for an active, healthy, and fulfilling lifestyle, which involves continuous positive interactions with the environment and embodied engagement in physical activities for life” [12]. Another example is Sport England, which recently published its own understanding of the concept, with PL covering individuals’ “own relationship with movement and physical activity throughout life” [13]. Although these definitions reveal some differences in the inclusion of certain domains (e.g. “social” in Australia or “perceptual” in China), central to PL is its grounding in philosophical assumptions [14, 15]. For instance, the core tenets underscore the inseparability of mind and body (monism). Therefore, PL detaches from one-sided (e.g. performance-centred) practices by highlighting more holistic and enriched experiences in physical activity contexts [16]. The tenets also stress the individual’s connection to their environment (existentialism) and the significance of personal experience in the sense of an individual’s unique journey (phenomenology) [17, 18]. Accordingly, the concept of PL incorporates an inviting and inclusive gesture toward each individual, stressing lifelong growth—irrespective of constitution, age and capabilities [19, 20].

Balancing fitness, skills, learning, enjoyment, knowledge acquisition and social experience, PL has the potential to act as an integrative interdisciplinary framework for physical activity promotion across all ages. Physical literacy can inform practices across the spectrum of physical activity and health, such as education, sport, public health, healthcare/promotion, rehabilitation, active recreation and community services/development. [21–24] The growing recognition of the concept in enhancing biopsychosocial health through physical activity is evidenced by its inclusion in influential global documents [25], such as the World Health Organisation’s Global Action Plan on Physical Activity 2018–2030 (GAPPA) and UNESCO’s Quality Physical Education Guidelines for Policymakers. For instance, the GAPPA repeatedly highlights PL and claims to “establish and reinforce lifelong health and PL, and promote the enjoyment of, and participation in, physical activity” [26]. Similarly, the UNESCO Quality Physical Education Guidelines for Policymakers awarded PL a central position within their document, emphasising that the “promotion of PL should then remain a key feature of any physical education curriculum throughout primary and secondary education” [27]. At the empirical level, PL appears to positively impact physical activity, positive human development, health and well-being [28–34].

### The Developmental and Global State of PL

Despite these developments favouring adoption, the PL field faces several challenges. For instance, the inclusion of PL into important global guidelines often does not permeate to the national and local levels, thus creating inconsistencies between national and international documents [35, 36]. Moreover, researchers do not fully acknowledge the theoretical foundations of PL and adjust these pragmatically to their purposes [37]. Conceptual critiques address the ambiguous definitions, contest some theoretical foundations, and raise concerns over exaggerated claims [4, 38, 39]. Assessments differ largely in psychometric quality and do not always align to the conceptual core of PL [40, 41]. There is also a dearth of assessments suitable for use at scale and with adults [35, 42, 43]. Practitioners voice difficulties in understanding the conceptual and philosophical foundations of PL, which hampers its appropriate delivery in settings that promote physical activity [44–46]. Programmes inspired by PL demonstrate low quality and tend to undermine holistic experiences that would sustainably attach individuals to self-initiated physical activity [47]. Moreover, there are limited data on the implementation of PL (e.g. via monitoring) into research, practice and policy, especially from low-and-middle income countries [36, 48].

In summary, the PL field has developed remarkably since its resurgence in academic discourse approximately two decades ago [49]. However, these challenges prevent further progress in the field. The challenges outlined above should be systematically targeted to ‘defend’ the core characteristics of PL, retain its acceptance and momentum, and enable further progress and innovation to better harness its potential for holistic personal development, health and well-being. Two articles in the past have incorporated prospective visions [50, 51], but the field developed rapidly in the last 10 years, and these initiatives should surpass the perspective of single researchers for greater impact. Consequently, the PL field can benefit from a broadly consented roadmap with a future-oriented vision [52] that: (i) embodies an interdisciplinary mindset; (ii) encompasses a range of target settings and applications (e.g. public health, physical education, sport, healthcare/promotion, rehabilitation, active recreation, and community services/development); (iii) unifies differing cultural understandings and progress; (iv) tolerates different paradigms [53]; (v) dissolves competing and conflicting networks [6, 54]; (vi) transcends the status of countries working in an isolated and separated manner from each other; and (vii) simultaneously considers research, practice and policy.

### Goal and Use of the Article

We endeavour to answer the following questions for research, practice and policy: Which *goals* can be defined for the future development of the field? Which *principles* must be considered, given the specific features of the PL concept? Which concrete *actions* would benefit this development? Which *pathways* offer the best opportunity to move the PL field forward? Accordingly, the goal of the present study was to define goals and principles (asking the future-related question *what* is needed?) as well as actions and pathways (asking *how* this can be done?) to effectively advance the PL field worldwide. In line with a progressive claim, the identified solutions will inform a Global Physical Literacy (GloPL) Action Framework. Specifically, a consensus statement will be developed to guide the articulation and dissemination of the agreed recommendations. The GloPL Action Framework will serve as a comprehensive tool to navigate the complex landscape of PL in the future.

## Methods

### Study Design and Overview

**Fig. 1:**
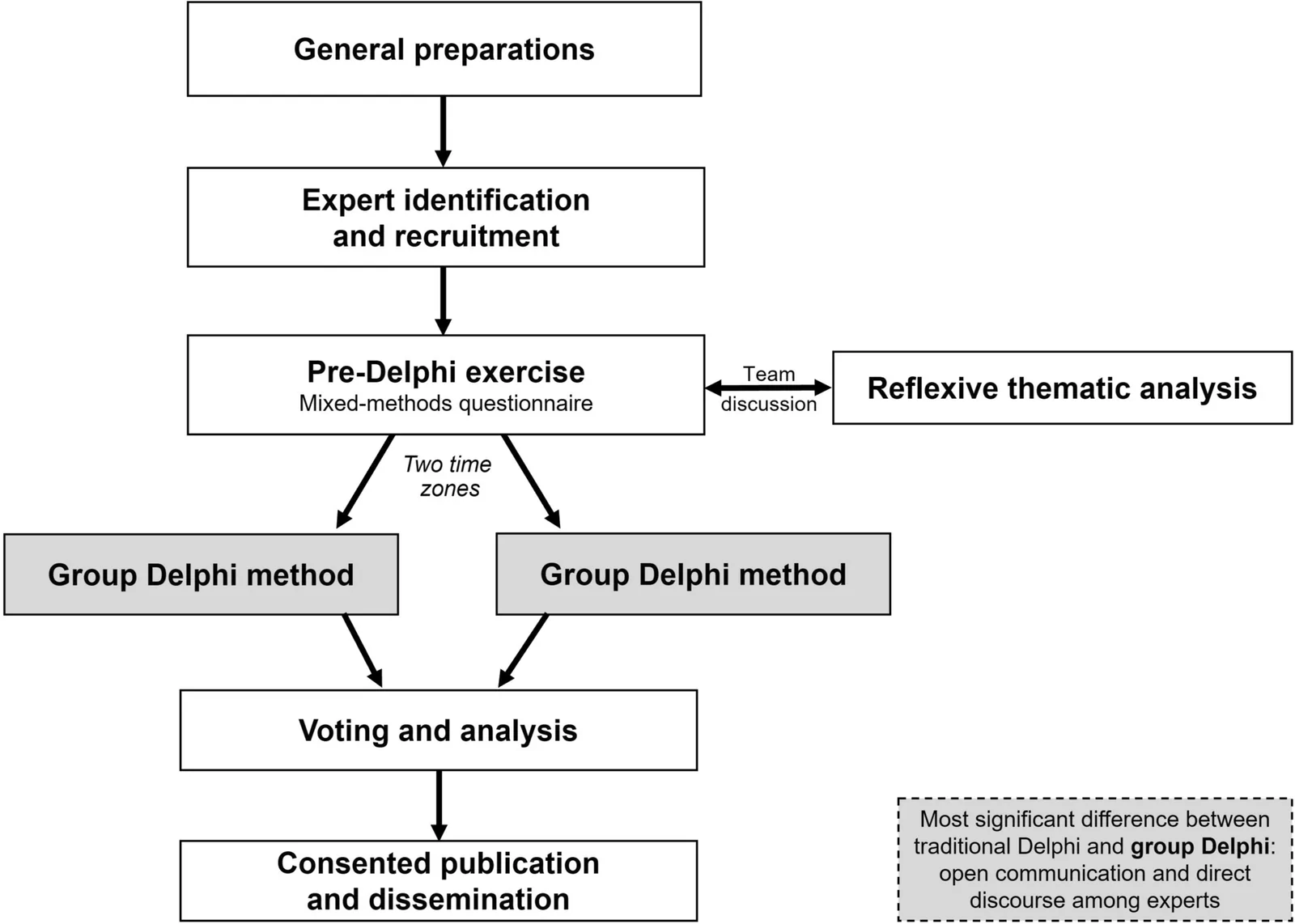
Entire process of the group Delphi method

Capitalising on the benefits of collective intelligence and distributed cognition, this study employed a group Delphi technique with global representation to (a) define the goals, principles, actions and pathways that significantly advance the PL field and (b) develop a GloPL Action Framework [55]. Unlike traditional Delphi methods that prioritise anonymity [56], this approach fostered open communication among experts and focused on the advantages of direct discourse. The group Delphi process was conducted online and comprised the following distinct phases [57, 58]: (a) general preparations (including literature review and survey design); (b) expert identification/recruitment; (c) a pre-Delphi exercise with qualitative analysis of responses; (d) group Delphi discussion and prioritisation of key themes; (e) voting and data analysis; and (f) dissemination of the findings via a common statement with the GloPL Action Framework. In summary, this group Delphi process involved three data sources for analysis (steps c/d/e). The combination of inductively generated qualitative data and subsequent quantitative data (voting) qualifies the present study as an exploratory sequential mixed-methods design [59, 60]. The entire group Delphi process, including all pre-defined decisions, was prospectively communicated in a study protocol [52] and is summarised in Fig. 1. Ethics approval for this study was granted by the Human Ethics Advisory Group Health at Deakin University (approval number HEAG-H 06_2024). All representatives provided informed written consent to participation and data collection. The reporting of the findings adheres to the guidance on Conducting and REporting DElphi Studies (CREDES; see Electronic Supplementary Material [ESM] for details) [61].

### Recruitment of Representatives

The selection of participants for this study was guided by three primary objectives, resulting in three different representation groups. First, the GloPL Action Framework aimed for *global representation* of experts to account for differential progress of PL worldwide. While acknowledging stronger progress of PL in North America, Europe and Australia [9, 35, 62], the selection process also aimed to avoid neglecting other regions of the world, including Africa, Latin America and the Caribbean, and Asia. Consequently, significant effort was dedicated to recruiting experts from countries with few PL reports. Second, we specifically included experts representing seven *themes of special interest* to address well-known gaps within the PL field [52]: older adults, climate change and sustainability, socioeconomic status and health inequalities, people with disabilities, indigenous perspectives, environmental design and practice translation (the application of PL principles into real-world settings). Third, recognising the importance of an effective dissemination of the framework, this study proactively involved representatives from *societies* within the broader physical activity and health landscape throughout the framework development. The society representatives integrated their external perspective into the consensus process without needing (through their roles) to contribute to idea generation and voting. For the purpose of this study, we operationalised an expert as a person who has considerable *knowledge* and/or *experience* with PL, and/or has the potential to significantly influence PL (e.g. via research, regulations, guidelines or policy) in their country, region or professional networks [63]. We defined the knowledge and experience status through thematically relevant research output in both quantity and quality (especially in the previous 5 years), coupled with a visible presence on websites (e.g. in societies, documents, practical initiatives), or in workshops. In addition to these primary criteria defining ‘expertise’, the following secondary considerations informed expert selection: (i) diversity of intellectual backgrounds and ‘schools of thought’ [54]; (ii) representation across application fields, including physical education, sport, public health, healthcare/promotion, rehabilitation, active recreation and community services/development; (iii) coverage of the entire lifespan; and (iv) inclusion of individuals with practical experience translating PL into real-world settings. Reflecting the multi-faceted nature of PL research and application, expert heterogeneity was prioritised over homogeneity [63].

### Pre-Delphi Exercise

After expressing initial interest (via e-mail) in joining the study as an expert (88.2% positive responses), the potential participants received a 2-min introductory video outlining the group Delphi process. Upon consenting to participate in the study, participants were provided with a two-part questionnaire. This questionnaire was tailored to the three representation groups. It began with open and multiple-choice questions about the individual participant (including represented country/society, area and duration of expertise, primary target group of activities and application field) as well as the perceived relevance of PL within their country or organisation. The second part of the questionnaire invited participants to share their opinions and ideas on advancing the PL field, encouraging them to consider research, practice and policy. Aligned with the study’s objectives, this section explored goals and principles (“What is needed?”) in the first question, and actions and pathways (“How could the goals or principles be addressed?”) in the second question. These open-ended questions allowed for free responses, but the participants were asked to limit the ideas to one page (approximately 400–500 words) per question. Participant reports of the two open-ended questions were analysed using Braun and Clarke’s six-step procedure for reflexive thematic analysis with the goal of hierarchically structuring the ideas and mapping these to different, yet interlinked, themes [64]: (1) familiarisation with the reports; (2) generation of initial codes; (3) identification of themes; (4) review and refinement of themes; (5) definition and labelling of themes; and (6) production of the final report. We explicitly permitted the assignment of ideas to multiple themes, if necessary. Further extending the qualitative position of this work, we emphasised the inductive (rather than deductive) nature of identifying structures within the material and undertook the coding at both semantic and interpretative levels [65] to uncover the contextually embedded meaning expressed by the representatives. The third author (AJ) independently performed steps 1–3, while the first author (JC) familiarised himself with the reports (step 1) to critically discuss and revise the themes in step 4. At this point, we highlight the non-linear nature of the process [65], as the two researchers undertook four iterations between steps 2 and 4 before reaching a comprehensive version of the qualitative analysis. Subsequently, this version was discussed within the coordinating team (first five authors) and was slightly refined again. The authors conducted the analysis of the Pre-Delphi exercise in NVivo, Version 14.23.3.

### Group Delphi Meetings

Based on the ideas from the group Delphi exercise, the coordinating team defined critical topics for discussion. Topics with a substantial number of codes (*n* ≥ 7), without conflicting or incompatible positions, and with a precisely defined message did not require debate and were directly included in the framework. Instead, priority for discussion was given to topics with (a) a smaller number of codes to provide a forum for unconventional, yet potentially innovative ideas, (b) conflicting and incompatible positions to forge a clear direction for the field and (c) the need to precisely define the suggestions due to its impact (e.g. careful consideration of ‘can’, ‘should’ or ‘must’ messages).

The topics were debated in three face-to-face online meetings (each 120 min long) facilitated via Zoom Version 5.16 (Zoom Video Communications, San José, CA, USA). We introduced all topics via presentation slides outlining the pre-Delphi results and raised specific questions to stimulate discussion. Because of the global coverage of representatives, we offered two different options for participation in the discussions: one discussion suitable for the Pacific and American time zones, and one meeting suitable for the European, African and Asian time zones (although participants were not restricted to their time zone and could also switch between the two options). The first author (JC) moderated all meetings, with the second (LB) and third authors (AJ) assisting where necessary. The participants could make verbal contributions or written comments (using Zoom’s chat function) at any discussion stage, which were read out in the meeting by the moderator. All meetings were recorded to analyse the meetings retrospectively and make the discussions accessible for all representatives who were unable to attend a meeting. Likewise, the representatives were always given the opportunity to provide feedback or seek assistance (e.g. regarding language-related challenges) outside the official sessions.

### Voting Procedure

All discussion topics concluded with formal voting to derive consensus recommendations for the GloPL Action Framework. The moderator first provided an overview of the options expressing the consideration or non-consideration of themes within the final framework. Afterward, the technical assistant of the meeting (AJ) opened a pre-prepared poll for anonymous voting on the preferred option. Although in the published study protocol we initially proposed to use Qualtrics (Provo, Seattle, WA, USA) [52], we later decided to use the Zoom embedded polling to avoid confusion from changing between software. In the first round of formal voting, at least two thirds (66.7%) of all voters had to agree with one statement for its final inclusion in the framework [66]. All statements below a positive agreement value of 25% were directly excluded. If the agreement rate was between 25 and 66.7%, the coordinating team met between the different meetings to find harmonising solutions across different options. These harmonising solutions were generated after reviewing the video recordings and examining in detail the distributions of the votes of the first round. The coordinating team calibrated the wording of the revised statements to proportionally reflect the degree of consensus for each controversial topic. Accordingly, topics with lower levels of agreement (a more split voting pattern) required more significant conceptual compromises to bridge the diverging viewpoints. In no case was the same statement submitted to second-round voting without modification. In the next meeting, these harmonising solutions were then presented and subjected to a second round of voting where the final decision was based on a simple majority (i.e. inclusion of themes with agreement ≥ 50% and exclusion of themes with agreement < 50%). However, we also gave representatives the opportunity to vote offline (via an exported document) if they were not able to attend a meeting. We used *χ*^2^ tests to assess whether the voting pattern differed significantly between the Pacific/American time zone round, the European/African/Asian time zone round and the offline voters.

## Results

### Representatives

We recruited 59 experts from 46 countries (geographical representatives). In line with the intended continental coverage [52], the representatives included 21 experts from Europe, 14 from Asia, 10 from North/Middle America, 6 from Oceania, 5 from Africa and 3 from South America. The identified experts, including their substitutes, from two regions did not respond, leaving these regions (Central West Africa, North of South America) unrepresented. Instead, we recruited one additional representative from the Southern part of Africa and one from the Iberian region, given Spain’s recent strong PL attention [67]. Moreover, we recruited exactly one individual for each of the seven themes of special interest, and 24 individuals from 19 societies. Further details on the representatives can be accessed in the ESM.

### Pre-Delphi Exercise

A total of 49 geographical representatives (83.1%), 13 society representatives (56.5%) and 7 representatives of special thematic interest (100%) submitted the Pre-Delphi exercise. The two coders extracted a total of 849 codes, which were (through interpretation) hierarchically bundled into three levels: themes (lowest level), overarching themes (medium level) and meta-themes (highest level). The five meta-themes included: advocacy (*n* = 351 codes), practice (*n* = 88 codes), education (*n* = 148 codes), assessment (*n* = 59 codes) and research (*n* = 203 codes). A visualisation of the themes, along with the hierarchical structure, can be accessed in the ESM. As part of the analysis, we also identified two topics with conflicting recommendations across individuals. First, while some experts reflected the importance of deriving a shared understanding of PL at the global level, other experts located this necessity (prioritising cultural sensitivity) at the national level. Second, while some experts highlighted that in terms of assessments, the PL field requires more standardised and validated instruments, others stressed that the field requires more unstandardised and non-linear methods.

### Group Delphi Meetings

Based on the reflexive thematic analysis, a total of 19 topics did not require further discussion in any of the group Delphi meetings and were directly included into the framework; these topics received a substantial number of codes, did not contain conflicting or incompatible positions, and allowed for formulating a precisely defined message (see priorities a–c in Sect. 2.4). An overview of these topics is given in Table 1 (sorted by meta-themes).

**Table 1:** Topics for direct inclusion in the Global Physical Literacy (GloPL) Action Framework

Topic no.	Topic	Assigned meta-theme	Number of codes
1	Improve awareness and understanding of PL among stakeholders and in the community	Advocacy	121
2	Need for cross-sector collaborations	Advocacy	23
3	Develop resources for practical facilitation and monitoring systems	Practice	28
4	Design and run more PL programmes	Practice	26
5	Integrate PL into the community (e.g. community sport, recreation sector, public events)	Practice	13
6	Leverage technology to promote PL	Practice	10
7	Craft environments and infrastructure in line with PL	Practice	9
8	Strengthen continuous professional development for different professions	Education	39
9	Design tools to assess PL across the lifespan	Assessment	12
10	Design tools in other languages	Assessment	12
11	Generate data on PL at the population level	Assessment	11
12	Use collaborative methods	Research	20
13	Extend interdisciplinarity	Research	11
14	Extend the study of methods to promote the uptake of research into routine practice (implementation science)	Research	8
15	Encourage participant voice	Research	7
16	Emphasise practical applications	Research	18
17	Emphasise translational research	Research	16
18	Target evidence gaps and better communicate the evidence base	Research	27
19	Better include First Nations perspectives and groups/countries with low socioeconomic status	Research	18

Note: these topics received a substantial number of codes, did not contain conflicting or incompatible positions, and allowed for a precisely defined message

*PL* physical literacy

The coordinating team (first five authors) selected 15 crucial topics across all five meta-themes for discussion and voting within the group Delphi meetings. Table 2 summarises these topics, including the durations of the corresponding discussions. Each meeting (for the timing, see ESM) started with an introduction and overview of the goals and steps of the project, and ended with an announcement of the topics for the next meeting. The attendance across all three representation groups was between 63.3% (*n* = 57) and 71.1% (*n* = 64) for all three meetings.

**Table 2:** Topics discussed in the group Delphi meetings

Topic no.	Topic	Meta-themes^a^	Reason for entering the 15 priority topics	Timepoint of discussion (meeting number)	Time for discussion (in minutes)
1	Global definition of physical literacy	Advocacy, research	B	1	30
2	Assessment of physical literacy	Assessment	B	1	20
3	Professional sport	Advocacy	A, C	1	7
4	Global agenda of sport	Advocacy	C	1	7
5	Differences between physical literacy and physical education	Advocacy	A	1	10
6	Sustainable development goals	Advocacy	A, C	2	15
7	Health literacy	Advocacy	A	2	10
8	Funding/investment	Practice, advocacy	C	2	15
9	Physical education curricula	Education	C	2	15
10	Physical activity and health nationally	Advocacy	C	2/3	10
11	Sport nationally	Advocacy	C	3	10
12	Dedicated society and network	Research, advocacy	C	3	5
13	Regular conferences	Research, advocacy	A, C	3	5
14	Academic journal	Research, advocacy	A	3	5
15	Education principles	Education, practice	C	3	15

^a^Some topics derived were the result of the assignment to multiple meta-themes

Reason A: small number of codes: provide a forum for rarely mentioned ideas, Reason B: conflicting and incompatible position to provide a clear direction for the field, Reason C: impactful decision, need to precisely define the message

### Voting

The participation rate for the surveys, which referred to the geographical representatives and representatives of special thematic interest [52], varied between 66.7% (*n* = 44) and 89.4% (*n* = 59), implying that the minimum response rate defined in the study protocol (66.7%) was fulfilled for all surveys. The representatives achieved direct consensus (≥ 66.7%) for 6 of 15 topics in the first voting round (Table 3):

**Table 3:** Results of the first round of voting

Topic and response options (in the presented order)	Absolute votes	Relative percentages
*TOPIC 1: GLOBAL DEFINITION OF PHYSICAL LITERACY*		
We need a consensus for a globally or universally accepted definition on physical literacy	5	8.6%
We do not need a definition on physical literacy on the global scale	1	1.7%
We need an obligatory core set of elements or principles, allowing for national or regional specificities	52	89.7%*
*TOPIC 2: ASSESSMENT OF PHYSICAL LITERACY*		
We need more standardized, validated assessments	0	0.0%
We need more personalized and non-linear assessments	2	3.8%
We should implement a “different lenses” message, highlighting different functions and purposes for different assessments	9	17.0%
We need fluidity: a varied approach to assessment, with a focus on quality	35	66.0%
We should consider a personal holistic reflection on our engagement in physical activity	7	13.2%
*TOPIC 3: PROFESSIONAL SPORT*		
We should explicitly exclude professional sports	3	5.4%
We should not explicitly exclude but also not specifically mention professional sports	32	57.1%
We should explicitly mention professional sports	21	37.5%
*TOPIC 4: GLOBAL AGENDA OF SPORT*		
We should advocate for putting physical literacy on the agenda of global sport	46	92.0%*
We should not advocate for putting physical literacy on the agenda of global sport	4	8.0%
*TOPIC 5: DIFFERENCES BETWEEN PHYSICAL LITERACY & PHYSICAL EDUCATION*		
We should not explicitly emphasise that stakeholders have to consider the differences between “physical education” and “physical literacy”	4	7.5%
We should explicitly emphasise that stakeholders have to consider the differences between “physical education” and “physical literacy”	49	92.5%*
*TOPIC 6: UNITED NATIONS SUSTAINABLE DEVELOPMENT GOALS*^*a*^		
We should specify to which sustainable development goals the physical literacy concept connects	36	62.1%
We should connect to the sustainable development goals but keep it abstract (this can be the task of future research)	19	32.8%
No, we should not connect physical literacy to the sustainable development goals	3	5.2%
*TOPIC 7: HEALTH LITERACY*		
Yes, we should advocate strengthening links between physical literacy and health literacy	28	50.0%
No, we should not advocate for strengthening links between physical literacy and health literacy	28	50.0%
*TOPIC 8: FUNDING/INVESTMENT*		
We should not include any statements about better funding	5	9.3%
We should only make indirect links without mentioning the word “funding”	22	40.7%
We should apply a ‘soft’ wording about better funding spread across the document	18	33.3%
We should be very explicit about better funding and provide a separate section	9	16.7%
*TOPIC 9: PHYSICAL EDUCATION CURRICULA* *Question about the explicit term*		
We should not advocate for harmonising physical education curricula with physical literacy	1	1.9%
The national physical education curricula should embody the pedagogical/didactical principles of physical literacy	9	17.3%
National physical education curricula should align with PL (e.g. through equivalent concepts in other languages)	21	40.4%
PL should be explicitly included in physical education curricula at all stages of education	21	40.4%
*Question about the reach*		
We should not mention physical literacy in the context of physical education curricula	0	0.0%
We should not define the reach in the framework	3	5.7%
We should define the reach on the national level	14	26.4%
We should define the reach on the national and the state/regional level	10	18.9%
We should define the reach at all levels of organisation (national, state, school)	26	49.1%
*TOPIC 10: PHYSICAL ACTIVITY AND HEALTH NATIONALLY* *Physical activity (promotion) guideline*		
We should not make the explicit call to include physical literacy in national physical activity (promotion) guidelines	0	0.0%
We should advocate for aligning national physical activity (promotion) guidelines with the principles of physical literacy	22	37.9%
We should advocate for explicitly including physical literacy in national physical activity (promotion) guidelines	36	62.1%
*National health policies*		
We should not make the explicit call to include physical literacy in health policy documents	3	10.7%
We should advocate for aligning national health policy documents with the principles of physical literacy	23	42.9%
We should advocate for explicitly including physical literacy in relevant health policy documents	31	46.4%
*TOPIC 11: SPORT NATIONALLY* *National Sport Policies*		
We should not make the explicit call to include physical literacy into national sport policies	1	2.1%
We should advocate that the vision/mission statements of major sport organisations should harmonise with physical literacy	26	55.3%
We should advocate for explicitly including physical literacy in the vision/mission statements of major sport organisations	20	42.5%
*Coaching Material*		
We should not make the explicit call to include physical literacy into coaching material	2	4.1%
We should advocate for harmonising coaching material with the concept of physical literacy	23	46.9%
We should advocate for explicitly including physical literacy into coaching material	24	49.0%
*TOPIC 12: DEDICATED SOCIETY AND NETWORK*		
Yes, we should include a statement that the field of physical literacy can benefit from establishing a dedicated society and network	24	50.0%
No, we should not include a statement that the field of physical literacy can benefit from establishing a dedicated society and network	24	50.0%
*TOPIC 13: REGULAR CONFERENCES*		
Yes, we should include a statement that the field of physical literacy can benefit from holding regular conferences on physical literacy	36	75.0%*
No, we should not include a statement that the field of physical literacy can benefit from holding regular conferences on physical literacy	12	25.0%
*TOPIC 14: ACADEMIC JOURNAL*		
Yes, we should include a statement that the field of physical literacy can benefit from establishing an academic journal on physical literacy	12	25.5%
No, we should not include a statement that the field of physical literacy can benefit from establishing an academic journal on physical literacy	35	74.5%*
*TOPIC 15: EDUCATION PRINCIPLES*		
Yes, I agree with the presentation of educational principles in line with physical literacy	37	84.1%*
No, I do not agree with the presentation of educational principles in line with physical literacy	7	15.9%

Note: percentages with as asterisk (*) surpassed the pre-defined threshold for sufficient consensus at the first stage (66.7%) and were considered as resolved

^a^For an overview of the United Nations Sustainable Development Goals, see https://sdgs.un.org/goals or, for the physical activity context, Salvo et al.[85]

Topic 2: The voting pattern differed between time zone rounds and offline voters, *χ*^2^(4) = 13.7,* p* = 0.008

Topic 8: The voting pattern differed between time zone rounds and offline voters, *χ*^2^(6) = 14.3,* p* = 0.027

Topic 12: The voting pattern differed between time zone rounds and offline voters, *χ*^2^(2) = 6.09,* p* = 0.048

Topic 14: The voting pattern differed between time zone rounds and offline voters, *χ*^2^(2) = 12.8,* p* = 0.002

*Topic 1—Global definition of PL:* instead of forcing a globally valid understanding, the group Delphi panel suggested that the field can benefit best from generating an obligatory core set of elements or principles, allowing for national or regional specificities (98.7%).

*Topic 4—Global agenda of sport:* the panel highlighted that stakeholders should congruently advocate for PL to be on the agenda of global sport (92.0%).

*Topic 5—Differences between PL and physical education:* although the designation “physical education” refers to the academic subject, external individuals appear to frequently equate or conflate this term with “physical literacy”. Therefore, it is imperative for the further development of the concept to explicitly emphasise the differences between these two terms (92.5%).

*Topic 13—Regular conferences:* the group Delphi underlined that the field of PL can benefit from holding regular conferences on PL to promote exchange on the topic (75.0%).

*Topic 14—Academic journal:* the establishment of a specific journal on PL does not move the field forward substantially. Instead of developing an insulated forum for academic exchange, the broad and interdisciplinary field of PL should continue to spread its discussions into the wide range of available journals and scientific disciplines (74.5%).

*Topic 15—Education principles:* The panel agreed that an action framework should clearly articulate educational principles in line with PL. The final principles, which will be explained in detail in the GloPL Action Framework, encompass the following short descriptions: ‘holistic learning and development’, ‘support and inclusion’, ‘lifelong journey’, ‘person in the centre’, ‘voice and choice’ and ‘work across sectors’ (84.1%).

Nine topics did not achieve direct consensus in the first round, with the coordinating team subsequently attempting to identify harmonising solutions between opposing sides for the second round of voting. The consensus decisions (≥ 50%) for the second round were (Table 4):

**Table 4:** Results of the second round of voting (if necessary)

Topic and response options (in the presented order)	Absolute votes	Relative percentages
*TOPIC 2: ASSESSMENT OF PHYSICAL LITERACY*		
Harmonising statement: “Physical literacy assessments or chartings should match their purposes. There is a place for standardized and psychometrically tested assessments (e.g. for monitoring population levels) as well as for qualitative approaches to account for the uniqueness of individual journeys.”		
No	0	0.0%
Basically yes, but I suggest amendments	10	17.5%
Yes, I fully agree	47	82.5%*
*TOPIC 3: PROFESSIONAL SPORT*		
Harmonising statement: “Physical literacy can serve as a vehicle across a range of fields (such as education, sport, care, recreation, health promotion, and community services) and be targeted towards those of differing levels within these settings (including professional).”		
Yes	32	54.2%*
No, and I suggest amendments	27	45.8%
*TOPIC 6: UNITED NATIONS SUSTAINABLE DEVELOPMENT GOALS*^*a*^		
We should keep the formulation abstract and not mention to which specific goals the physical literacy concept connects	16	34.0%
We should specify to which Sustainable Development Goals the physical literacy concept connects^b^	31	66.0%*
*TOPIC 7: HEALTH LITERACY*		
Harmonising statement: “There are overlaps between physical literacy and health literacy. However, the two concepts were developed for distinct purposes. Advocating for physical literacy can leverage on the well-known concept of health literacy, but physical literacy should not be equated with health literacy or portrayed as a sub-component of health literacy.”		
Yes, I agree	45	95.7%*
No, I do not agree	2	4.3%
*TOPIC 8: FUNDING/INVESTMENT*		
Question: Do you agree with our suggestion to only use indirect links (e.g. to maximize efforts”) and, if not avoidable, to prefer the word “investment” instead (e.g. sections on advocacy, practices)?		
Yes, I agree	44	93.6%*
No, I do not agree	3	6.4%
*TOPIC 9: PHYSICAL EDUCATION CURRICULA*		
Do you agree with our harmonising solution that the physical education curricula should align with the principles of physical literacy, that the term physical literacy (or relevant translations) should be used in education curricula, and that we mean all curricula at the national, state/regional, and local school levels?		
Yes, I agree	46	93.9%*
No, I do not agree	3	6.1%
*TOPIC 10: PHYSICAL ACTIVITY AND HEALTH NATIONALLY*		
Do you agree with our harmonising solution that we should advocate for explicitly including PL in national physical activity (promotion) guidelines and for aligning national health policy documents with the principles of PL?		
Yes, I agree	55	98.2%*
No, I do not agree	1	1.8%
*TOPIC 11: SPORT NATIONALLY*		
Do you agree with our harmonising solution that we should advocate for the strategies, policies, operations, and practices of national sport organisations to align with physical literacy and for the coaching material to harmonise with the concept of physical literacy?		
Yes, I agree	51	91.1%*
No, I do not agree	5	8.9%
*TOPIC 12: DEDICATED SOCIETY AND NETWORK*		
Do you agree with our harmonising solution that the field of physical literacy can benefit from the installation of specific working groups or special interest groups within existing societies or networks		
Yes, I agree	52	92.9%*
No, I do not agree	4	7.1%

Note: percentages with as asterisk (*) surpassed the pre-defined threshold for sufficient consensus (50.0%) at the second stage and were considered as resolved. The voting pattern did not differ between time zone rounds and offline voters for any of the second-round topics

^a^For an overview of the United Nations Sustainable Development Goals, see https://sdgs.un.org/goals or, for the physical activity context, Salvo et al. [85]

^b^In addition to this response, the participants were given the opportunity to select among the list of 17 sustainable development goals

*Topic 2—Assessment of PL:* in line with a tolerant stance on assessment, the group Delphi panel suggested that any PL assessment or charting should match its purposes; there is a place for standardised and psychometrically tested assessments (e.g. for monitoring population levels) as well as for qualitative approaches to account for the uniqueness of individual journeys (82.5%).

*Topic 3—Professional sport:* reflecting on the role and potential of ‘professional sport’, the group Delphi panel decided to list professional sport within the action framework only in brackets among the fields where PL might impact (54.2%).

*Topic 6—United Nations Sustainable Development Goals:* the representatives anticipated that stronger connections between PL and Sustainable Development Goals (SDGs) can move the field forward, and that the goals should be specified (66.0%). Participants who expressed confidence to specify the most important SDGs (*n* = 22; see ESM for further details) highlighted the function of PL in fostering good health and well-being (SDG-3; 100%); quality education (SDG-4; 90.9%); sustainable cities and communities (SDG-11; 63.6%); gender equality (SDG-5; 59.1%); and reduced inequalities (SDG-10; 59.1%).

*Topic 7—Health literacy:* after an intense debate about the relationship between PL and health literacy, the following statement finally found support by the group Delphi panel (95.7%): “There are overlaps between PL and health literacy. However, the two concepts were developed for distinct purposes. Advocating for PL can leverage on the well-known concept of health literacy, but PL should not be equated with health literacy or portrayed as a sub-component of health literacy”.

*Topic 8—Funding/investment:* the panel derived the suggestion that the framework should only use direct links to advocate for increased resources for the translation of PL into real-world action and for research, preferring the word “investment” in this context (93.6%).

*Topic 9—Physical education curricula:* physical education curricula at the national, state/regional and local school levels should align with the pedagogical principles of PL and the term itself (or relevant translations) should be used in education curricula (93.9%).

*Topic 10—Physical activity and health nationally:* the panel endorsed the statement that broad national health strategies must align with the principles of PL and, importantly, that PL must be explicitly included in the physical activity (promotion) guidelines (98.2%).

*Topic 11—Sport nationally:* the GloPL Action Framework should recommend that national sport organisations and associations must align their strategies, policies, operations and practices with PL, and that the educational material for current and future coaches must harmonise with the concept of PL (91.1%).

*Topic 12—Dedicated society and network:* lastly, the group Delphi panel agreed that the field of PL can benefit from the installation of specific working groups or special interest groups within existing societies or networks (92.9%).

## Discussion

**Fig. 2:**
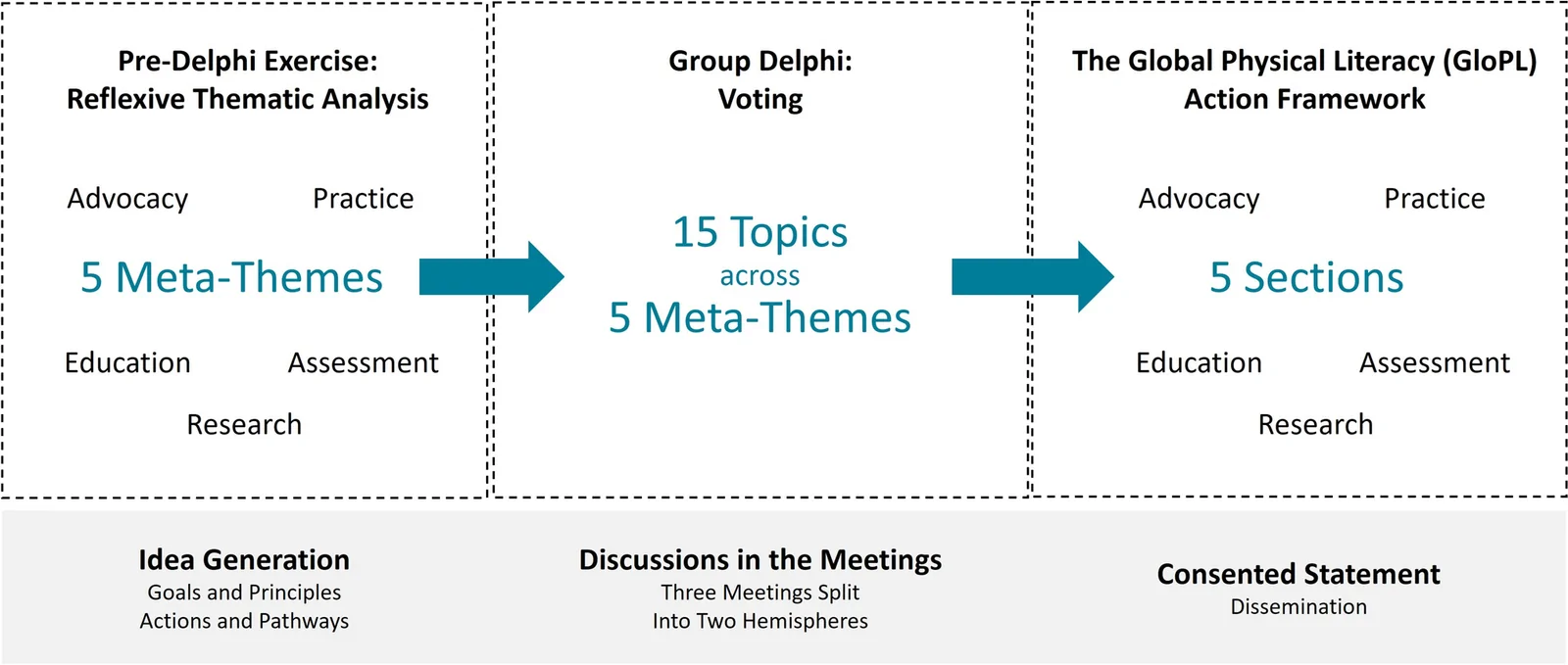
Five meta-themes as the consistent element across the entire process toward the development of the Global Physical Literacy (GloPL) Action Framework

The goal of the present study was to identify goals, principles, actions and pathways that can significantly advance the concept and its applications worldwide. Capitalising on a group Delphi method with three representation groups, the involved experts generated crucial ideas for an action framework categorised under ‘advocacy’, ‘practice’, ‘education’, ‘assessment’ and ‘research’. These meta-themes not only emerged during the pre-Delphi exercise but also subsequently informed the 15 topics of the group Delphi process. Following a coherent process (Fig. 2), these meta-themes will also drive the structure of the GloPL Action Framework.

The group Delphi process resolved directional decisions for further development of the PL field. For instance, the panel achieved consensus for the need of a universally valid definition of PL (Topic 1) and pathways of future assessments (Topic 2). While the qualitative analysis of the independent idea generation revealed conflicting standpoints for both topics, the face-to-face meetings resulted in clear suggestions for future work. For instance, researchers have extensively discussed how to deal with differing conceptualisations and definitions. Whilst some researchers saw a solution in structured consensus processes working toward stronger coherence in definitions [68], others called for stronger tolerance and clear articulation of the position adopted [69, 70], and others more recently questioned whether a uniform global definition is realistic and desirable at all [71]. The assembly of worldwide experts suggested that forcing a uniform definition would probably entail more harm than benefit by imposing an external understanding and by marginalising culturally embedded understandings (thus resulting in potential neglect of PL). However, to retain the integrity of the concept, the PL field can benefit from establishing a shared core set of elements or principles, allowing for specifications at the national and regional levels. In this regard, the consensus process identified synergistic solutions for enhanced stakeholder dialogue, such as the establishment of dedicated working groups or special interest groups within existing societies or networks (Topic 12) and the organisation of regular conferences (Topic 13). Likewise, reflections on the adoption of PL into physical education curricula (Topic 9), physical activity (promotion) guidelines (Topic 10) and national sport policies (Topic 11) constitute important opportunities to use the core set to specify the nationally most appropriate set of PL components.

Moreover, researchers have adopted differing positions regarding the assessment of PL, which range from the rejection of quantitative measurements [18, 72, 73] to the traditional application of psychometric criteria [41, 74, 75]. In this regard, the panel underlined that both qualitative methods and quantitative assessments have their value and place in research and practice, with the purpose and context (e.g. one-on-one conversation vs population monitoring) marking the decisive factors for their selection. Similarly, the process revealed that the advancement of the PL field is strongly linked to better advocacy at multiple levels (e.g. the global sphere of sport, national health promotion strategies, sustainability aspects). In summary, the outcome of these two discussion topics may express that PL has the potential to unfold across further regions and applications by dissolving narrow boundaries. The voting results illustrated that the experts coming from different disciplines and ‘schools of thought’ gained better mutual understanding through the discussion process. This resulted in a shift (despite anonymity) from more rigorous to more moderate positions. While the discussion process was insightful and resolved some key issues, it is crucial to underline that debate about the conceptual basis and the accountability of PL should not end with this framework [4, 38].

Apart from the present article, the GloPL Action Framework will be communicated on a specific website (see Declarations) in a statement document, released together with all geographical representatives, representatives of special thematic interest and the involved scientific organisations (after their external review/approval). The action framework defines policymakers, decision makers, practitioners in leadership roles (e.g. educators, coaches, health professionals, community managers), industry partners and researchers as its primary audience. To improve translation into practice [44–46], the GloPL Action Framework must include tangible messages and be presented in plain language. This transfer does not imply that the derived messages do not need a solid scientific foundation [76]. Rather, all main messages must be fully endorsed by the rigorous process and collaborative agreement amongst experts to meet the character of a consensually developed framework supported by scientific standards. However, the GloPL Action Framework is not an endpoint but might serve as a vital catalyst for the development of the PL field. To ensure the framework remains a dynamic instrument, the assembly proposes a formal review and update of the GloPL Action Framework in 5 years (2031). Stronger collaboration with regional networks, such as the African Physical Activity Network (AFPAN) and the South American Physical Activity and Sedentary Behaviour Network (SAPASEN), could help strengthen the global coverage and diversity of perspectives in future updates of the framework. Future updates should also strengthen the structured involvement of practitioners and implementation actors across sectors and regions. This 5-year timeline is strategically chosen to align with the conclusion of the World Health Organization’s Global Action Plan on Physical Activity 2018–30, allowing the framework to respond to the next generation of global health policy. The upcoming framework requires monitoring and evaluation regarding its acceptance, use, and actual implementation across different countries and sectors. The broad field of physical activity has developed policy- and behaviour-related evaluations [1, 77], with the proposed methods potentially inspiring similar endeavours for the PL field. For instance, the framework interest can be evaluated through citation metrics and document access on the website, while adoption can be monitored via the inclusion of PL in national physical activity guidelines, the alignment of coach education with PL principles and the formalisation of PL networks within established societies. However, translating such institutional adoption (and how these sectors interact to create enhancing and inhibiting factors) into practice requires navigating the political economy of each sector. Structural barriers, such as competing funding priorities (e.g. high-performance sport models) as well as traditions and standardised testing in education, often constrain the implementation of PL principles [21, 35]. To overcome these hurdles, the abstract goals of the GloPL Action Framework must be adapted to specific resource contexts. In countries with few resources, curriculum reform (Topic 9) could focus on low cost-equipment, culturally situated movement practices and realigned teacher education, while population-level assessment (Topic 2) could integrate a few items into existing population health surveys instead of implementing stand-alone psychometric tools. The operationalisation of these recommendations will require context-sensitive adaptation across differing political, cultural and resource conditions. Acknowledging these political and economic conditions ensures that the framework serves as a flexible catalyst rather than a rigid theoretical ideal.

Despite the systematic and coherent process, heterogeneity of involved experts and inclusion of representatives from diverse societies, we acknowledge the following limitations of our study. First, cultural and linguistic biases have co-influenced the group Delphi process. We observed after the first meeting that representatives from non-Western and non-Anglophone countries participated less in the discussions. To better overcome this barrier, we allowed and encouraged the participants to use the chat function during the meetings, and, in fact, diverse opinions were better expressed. People from English-speaking countries who were at risk of dominating the discussion process were asked to wait to allow others to add in their thoughts from the chat. However, the English-dominant process may have constrained the conceptual vocabulary to Western paradigms, potentially under-representing holistic or relational movement meanings that are differently captured in other linguistic traditions. To mitigate such biases, future global endeavours should more deliberately encourage the utilisation of asynchronous feedback (including options for anonymous variants). Similarly, experts from countries with low PL activities accepted invitations to this global project less frequently. Although we invested increased effort in sending kind reminder messages and in identifying alternative experts, there were two representation gaps that could not be filled (Central West Africa, and the North of South America). This highlights the structural challenge that in regions where PL is less visible, identifying representative experts is difficult. The absence of representatives from these two regions may have afforded less prominence to perspectives centred on communal movement practices and informal community-integrated play that flourish outside the institutionalised structures common in Western and Anglophone countries. More research in low- and middle-income countries is needed to broaden the global understanding of movement [78]. Future iterations should proactively seek these voices—perhaps by searching under the broader ‘physical activity’ frame for these areas—to further balance the framework’s emphasis on formal sectors with decolonial and indigenous movement philosophies [79]. Relatedly, the coordinating team, albeit diverse in nationality, was largely composed of actors from high-income countries. Second, the moderator (first author [JC]) had a decisive position in navigating the discussions and coordinating communication. To reduce this single-person influence, the coordinating team discussed all results together and took all directive decisions (including the definition of crucial topics and identification of harmonising suggestions) as a group. Third, because of the coverage of representatives across the world, we had to divide the online meetings by continents into two discussion rounds. Although a simultaneous discussion with all representatives would have been desirable, there was no alternative for this solution to ensure proper attendance and only four out of 24 voting patterns differed significantly by time zones. Fourth, although we reserved one position for translation into practice (representatives of special thematic interest) and one meta-theme was dedicated to ‘practice’, the selection criteria prioritised (global) output visibility, leading to a panel predominantly composed of academics. Consequently, the current emphasis may skew toward a theoretical and strategic consensus rather than immediate operational grassroots perspectives. Fifth, it would have been beneficial to extend the discussion time for more complex topics (e.g. Topic 6 *–* SDGs). However, we have already chosen an interactive Delphi method with open communication, with the number and duration of sessions at the upper limits of recommendations to prevent experts’ dropout from too intense involvement [80, 81].

## Conclusions

This group Delphi study brought together a large range of perspectives across the world to develop the first GloPL Action Framework. The development of this framework explicitly integrated research, policy, and practice aspects and may guide future activities across a range of fields, such as education, sport, public health, healthcare/promotion, rehabilitation, active recreation and community services/development [21–23]. While the framework is designed for multi-sectoral application, sectors with established institutional structures—such as formal education and organised sport—may be particularly conducive to effective implementation through existing systemic levers. In summary, the GloPL Action Framework can make a substantial contribution to narrowing the gap between science/policy and practice in the realm of physical activity, exercise and sport [82–84] by providing a roadmap for strengthening quality experiences. To strengthen the adoption of the action framework, the group Delphi study has deliberately incorporated geographical representatives to facilitate the spread of the framework and its implications across different global regions. Additionally, the inclusion of representatives of societies aimed to leverage existing network structures and establish communication channels. Therefore, the entire panel will convene for a dedicated workshop (see ESM) to promote the coordinated dissemination of the first GloPL Action Framework. This phase will explicitly include recommendations for countries to operationalise the framework at the national level by working with policy actors and practitioner organisations across different governance levels (e.g. national, state-wide, regional and communal), such as teacher federations, health ministries, sport associations, and community institutions to maximise the chance for local adaptation and real-world implementation. Future research can take an assisting role in solving conceptual challenges (Topics 1/4/5/6/15), advancing assessment solutions (Topic 2), informing policy guides (Topics 9/10/11) and providing adequate platforms for communication (Topics 12/13/14).

## Supplementary Information

Below is the link to the electronic supplementary material.

Supplementary file1 (DOCX 680 KB)

## Declarations

### Availability of Data and Material

The Global Physical Literacy (GloPL) Action Framework, including the main document with respective translations and the consortium, is available on the following website: https://blogs.deakin.edu.au/glopl/.
