# Supplementary material for: The Global Physical Literacy Action Framework: Process and Outcomes
Source: Sports Med. 2026 Aug 8;56(11):2873–92. doi: 10.1007/s40279-026-02485-6 (PMC13672500; doi:10.1007/s40279-026-02485-6)
Supplement: Supplementary file 1 [file 40279_2026_2485_MOESM1_ESM.docx]

**Electronic Supplementary Material**

*Article:* The Global Physical Literacy Action Framework: Process and Outcomes

*Journal Name:* Sports Medicine

*First/corresponding Author:* Dr. Johannes Carl (Deakin University, Institute for Physical Activity and Nutrition, Geelong, Victoria, Australia) – E-Mail Address: j.carl@deakin.edu.au

*Other Authors:* Dr. Johannes Carl (Deakin University, Institute for Physical Activity and Nutrition, Australia), Prof. Lisa Barnett (Deakin University, Australia), Dr. Alethea Jerebine (Deakin University, Australia), Dr. Emiliano Mazzoli (Deakin University, Australia), Prof. Alexandre Mouton (University of Liège, Belgium), Prof. Jaime Barratt (Brock University, Canada), Dr. Peter Elsborg (Copenhagen University Hospital, Denmark), Nigel Green (International Physical Literacy Association, United Kingdom), Prof. Marlen Niederberger (University of Education Schwäbisch Gmünd, Germany), Prof. Raimond Kim-Wai Sum (Chinese University of Hong Kong, China) on behalf of the GloPL Consortium

*Electronic Supplementary Material 1.* Research Checklist - The Guidance on Conducting and REporting DElphi Studies (CREDES) [61].

| **No.** | **Short description** | **Elaboration** | **Location in the article** |
| --- | --- | --- | --- |
| 1 | Justification | The choice of the Delphi technique as a method of systematically collating expert consultation and building consensus needs to be well justified. When selecting the method to answer a particular research question, it is important to keep in mind its constructivist nature. | Page 39 |
| 2 | Planning and process | The Delphi technique is a flexible method and can be adjusted to the respective research aims and  purposes. Any modifications should be justified by a rationale and be applied systematically and rigorously. | Study protocol [52], pages 29, 30 and 33 |
| 3 | Definition of consensus | Unless unreasonable due to the explorative nature of the study, an a priori criterion for consensus  should be defined. This includes a clear and transparent guide for action on (a) how to proceed with certain items or topics in the next survey round, (b) the required threshold to terminate the Delphi process and (c) procedures to be followed when consensus is (not) reached after one or more iterations. | Study protocol [52], pages 33 and 34 |
| 4 | Informational input | All material provided to the expert panel at the outset of the project and throughout the Delphi process should be carefully reviewed and piloted in advance to examine the effect on experts’ judgements and to prevent bias. | Pages 31 and 32;  Supplementary files 4-8 |
| 5 | Prevention of bias | Researchers need to take measures to avoid directly or indirectly influencing the experts’ judgements. If one or more members of the research team have a conflict of interest, entrusting an independent researcher with the main coordination of the Delphi study is advisable. | Pages 32, 33, 39 and 40 |
| 6 | Interpretation and processing of results | Consensus does not necessarily imply the ‘correct’ answer or judgement; (non)consensus and stable disagreement provide informative insights and highlight differences in perspectives concerning the topic in question. | Pages 35-39 |
| 7 | External validation | It is recommended to have the final draft of the resulting guidance on best practice reviewed and approved by an external board or authority before publication and dissemination. | Page 39 |
| 8 | Purpose and rationale | The purpose of the study should be clearly defined and demonstrate the appropriateness of the use of the Delphi technique as a method to achieve the research aim. A rationale for the choice of the Delphi technique as the most suitable method needs to be provided. | Pages 27 and 28 |
| 9 | Expert panel | Criteria for the selection of experts and transparent information on recruitment of the expert panel, socio-demographic details including information on expertise regarding the topic in question, (non)response and response rates over the ongoing iterations should be reported. | Pages 30, 31, 33 and 34 |
| 10 | Description of the methods | The methods employed need to be comprehensible; this includes information on preparatory steps  (How was available evidence on the topic in question synthesised?), piloting of material and survey instruments, design of the survey instrument(s), the number and design of survey rounds, methods of data analysis, processing and synthesis of experts’ responses to inform the subsequent survey round and methodological decisions taken by the research team throughout the process. | Pages 29-33 |
| 11 | Procedure | Flow chart to illustrate the stages of the Delphi process, including a preparatory phase, the actual ‘Delphi rounds’, interim steps of data processing and analysis, and concluding steps. | Figure 1, pages 32 and 33 |
| 12 | Definition and attainment of consensus | It needs to be comprehensible to the reader how consensus was achieved throughout the process, including strategies to deal with non-consensus. | Pages 33 |
| 13 | Results | Reporting of results for each round separately is highly advisable in order to make the evolving of consensus over the rounds transparent. This includes figures showing the average group response, changes between rounds, as well as any modifications of the survey instrument such as deletion, addition or modification of survey items based on previous rounds. | Tables 3 and 4, pages 33-37 |
| 14 | Discussion of limitations | Reporting should include a critical reflection of potential limitations and their impact on the resulting guidance. | Pages 39 and 40 |
| 15 | Adequacy of conclusions | The conclusions should adequately reflect the outcomes of the Delphi study with a view to the scope and applicability of the resulting practice guidance. | Figure 2, pages 37-41 |
| 16 | Publication and dissemination | The resulting guidance on good practice should be clearly identifiable from the publication, including recommendations for transfer into practice and implementation. If the publication does not allow for a detailed presentation of either the resulting practice guidance or the methodological features of the applied Delphi technique, or both, reference to a more detailed presentation elsewhere should be made (e.g. availability of the full guideline from the authors or online; publication of a separate paper reporting on methodological details and particularities of the process (e.g. persistent disagreement and controversy on certain issues)). A dissemination plan should include endorsement of the guidance by professional associations and health care authorities to facilitate implementation. | Pages 39-41, supplementary file 9 |

*Electronic Supplementary Material 2.* Effective recruitment of geographical representatives.

| **Continent** | **Country/region** | **Number of representatives** | | **Comment and/or included countries per region** |
| --- | --- | --- | --- | --- |
|  |  | **Planned (see  study protocol)^1^** | **Effective** |  |
| Europe | United Kingdom | 4 | 4 | “Motherland” of physical literacy, many activities, consensus statements, state-wide organization (England, Wales, Scotland, Northern Ireland) |
|  | Ireland | 1 | 1 | Progress in PL activities |
|  | German-speaking countries | 1 | 1 | Germany, Switzerland, Austria, Liechtenstein |
|  | France | 1 | 1 | Including Monaco |
|  | Italy | 1 | 1 | Including San Marino and Holy See |
|  | Iberian countries | 1 | 2 | Spain, Portugal, Andorra |
|  | Denmark | 1 | 1 | Non-anglophone country with considerable progress in physical literacy, own consensus |
|  | Northern regions | 2 | 2 | Sweden, Finland, Norway, Iceland, Greenland, Faroe Islands |
|  | Be-Ne-Lux countries | 1 | 1 | Belgium, Netherlands, Luxembourg |
|  | Central Europe | 1 | 1 | Czechia, Hungary, Poland, Slovakia, Slovenia |
|  | Baltic countries | 1 | 1 | Lithuania, Latvia, Estonia |
|  | Balkan countries | 2-3 | 2 | Albania, Bosnia and Herzegovina, Bulgaria, Greece, Kosovo, Montenegro, North Macedonia, Croatia, Serbia |
|  | Eastern Europe* | 1 | 1 | Ukraine, Moldova, Romania |
|  | Mediterranean countries | 0-1 | 1 | Cyprus, Malta |
| North and Middle America | Canada | 4 | 4 | Leading academic country, many practical initiatives related to physical literacy |
|  | United States of America | 3 | 3 | Large country with several academic and practical initiatives related to physical literacy |
|  | Mexico | 1 | 1 | Large country |
|  | Caribbean countries | 1 | 1 | Jamaica, Cuba, Trinidad and Tobago, Antigua and Barbuda, Bahamas, Barbados, Dominica, Dominican Republic, Grenada, Guyana, Haiti, Saint Kitts and Nevis, Saint Lucia, Saint Vincent and the Grenadines |
|  | Middle America mainland | 1 | 1 | Costa Rica, Panama, Nicaragua, Guatemala, Honduras, El Salvador, Belize |
| South America | Brazil | 1 | 1 | Large country |
|  | North | 1 | 0 | Colombia, Venezuela, Guyana, French Guiana, Suriname |
|  | West | 1 | 1 | Peru, Bolivia, Ecuador |
|  | South | 1 | 1 | Argentina, Chile, Uruguay, Paraguay |
| Asia | China | 2 | 2 | Large and populous country with many physical literacy initiatives, recent consensus process |
|  | India | 1-2 | 2 | Large and populous country with a growing number of physical literacy initiatives |
|  | Japan | 1 | 1 | Large country |
|  | East Asia | 1-2 | 2 | South Korea, Taiwan, North Korea, Mongolia |
|  | West Asia / Middle East | 2-3 | 2 | Türkiye, Bahrain, Kuwait, Oman, United Arab Emirates, Yemen, Armenia, Azerbaijan, Georgia, Iran, Iraq, Palestine, Syria, Saudi Arabia, Qatar, Israel, Jordan, Lebanon |
|  | Central Asia | 1 | 1 | Kazakhstan, Kyrgyzstan, Tajikistan, Turkmenistan, Uzbekistan |
|  | Hong Kong | 1 | 1 | Very strong academic progress |
|  | South-Central Asia | 1 | 1 | Pakistan, Afghanistan, Bhutan, Maldives, Sri Lanka, Bangladesh, Nepal |
|  | South-Eastern Asia | 2-3 | 3 | Brunei, Cambodia, East Timor, Indonesia, Laos, Malaysia, Myanmar, Philippines, Singapore, Thailand, Vietnam |
| Africa | Northern East | 1 | 1 | Eritrea, Sudan, Chad, Egypt, Libya |
|  | Northern West | 1 | 1 | Algeria, Morocco, Tunisia, West Sahara, Mauritania, Mali, Niger, Senegal, Gambia |
|  | Central West | 1 | 0 | Benin, Burkina Faso, Cape Verde, Ivory Coast, Ghana, Guinea, Guinea-Bissau, Liberia, Nigeria, Sierra Leone, Togo, Angola, Cameroon, Central African Republic, DR Congo, Equatorial Guinea, Gabon, Congo-Brazzaville, Sao Tome and Principe |
|  | Central East | 1 | 1 | Burundi, Comoros, Djibouti, Ethiopia, Kenya, Madagascar, Malawi, Mauritius, Mozambique, Rwanda, Seychelles, Somalia, South Sudan, Uganda, Zambia, Tanzania |
|  | Southern | 1 | 2 | South Africa, Lesotho, Botswana, Eswatini, Namibia, Zimbabwe |
| Australia and Oceania | Australia | 4 | 4 | Many academic and practical initiatives related to physical literacy |
|  | New Zealand | 1 | 1 | Own culture and understanding of physical literacy |
|  | Pacific islands | 1 | 1 | Fiji, Papua New Guinea, Solomon Islands, Vanuatu, Micronesia, Kiribati, Marshall Islands, Nauru, Palau, Cook Island, Niue, Samoa, Tonga, Tuvalu |

Note: The pre-defined number of representatives per region has been transparently described in the study protocol (see [52]).

*Electronic Supplementary Material 3.* List of the included number of academic societies.

| **Nr.** | **Abbreviation** | **Full name** | **Nominated number of representatives** | **Rationale** |
| --- | --- | --- | --- | --- |
| 1 | UNESCO (via Chairs) | United Nations Educational, Scientific and Cultural Organization | 2 | Umbrella organization for global education, physical education as an important subject, quality physical education guidelines for policymakers (has included physical literacy) |
| 2 | HEPA Europe | European network for the promotion of health-enhancing physical activity | 1 | Important network for physical activity and health, under the umbrella of the World Health Organization (“provide a forum for the advancement of health-enhancing physical activity research, policy and practice for better health and well-being” across Europe) |
| 3 | AIESEP | Association Internationale des Écoles Supérieures d'Éducation Physique | 1 | Important association at the intersection of physical education and health (“We promote effective physical and health education, physical activity and sport participation across the lifespan.”) |
| 4 | ISCA | International Sport and Culture Association | 1 | Important association for recreational sport and physical activity (“We empower organisations worldwide to enable citizens to enjoy their human right to move”) |
| 5 | EUPEA | European Physical Education Association | 2 | Important association for physical education (“Promote and defend physical education as an important educational area within the school system and the training of young people”) |
| 6 | ICSSPE | International Council of Sport Science and Physical Education | 2 | Important association in the broad field of sport science (“maintaining an inclusive perspective on the different disciplines of sport, sport science and physical education”) |
| 7 | FIEPS | Fédération Internationale d'Education Physique et Sportive | 1 | Important federation to promote physical activity in many spheres (“to promote, in all countries, the development of physical, educational, recreational and leisure activities, for the training of teachers”) |
| 8 | ASPA | Asia-Pacific Society for Physical Activity | 1 | Important society for physical activity (“to advance the science and practice of physical activity in Australasia through education and research”) |
| 9 | ACSM | The American College of Sports Medicine | 1 | Important society for sport medicine and exercise science (“advances and integrates scientific research to provide educational and practical applications of exercise science and sports medicine”) |
| 10 | ISPAH | International Society for Physical Activity and Health | 0* | Important society for physical activity and health (“To advance and promote physical activity as a global health priority through excellence in science (research), education, capacity building and advocacy”) |
| 11 | ISBNPA | International Society of Behavioral Nutrition and Physical Activity | 1 | Important society for changing physical activity behavior (“We stimulate and promote innovative and impactful research in behavioral nutrition and physical activity to improve human and planetary health and well-being worldwide”) |
| 12 | NASPSPA | North American Society for the Psychology of Sport and Physical Activity | 1 | Important society in the field of human physical activity behavior (“develop and advance the scientific study of human behavior when individuals are engaged in sport and physical activity; facilitate the dissemination of information […]”) |
| 13 | NASPEM | North American Society for Pediatric Exercise Medicine | 2 | Important society for the promotion of exercise in children and adolescents (“to promote exercise science, physical activity, and fitness in the health and medical care of children and adolescents”) |
| 14 | IMDRC | International Motor Development Research Consortium | 1 | Important network for motor development research (“To increase the visibility and impact of Motor Development research over the next decade through meaningful collaborations [… and to ensure that] all people reach their fullest potential to live an active and healthy life through movement”) |
| 15 | ACPES | ASEAN Council of Physical Education and Sport | 1 | Important society for physical education and sport in the ASEAN region (“to promote communication, interaction, and cooperation among ASEAN physical and sport education professionals and students in educational, scientific, and research institutions”) |
| 16 | SAPASEN | South American Physical Activity and Sedentary Behavior Network | 1 | Important society in South America for physical activity behavior (“To bring researchers and policymakers across the South American continent together to […] promote evidence-based strategies on physical activity and sedentary behavior”) |
| 17 | SHAPE America | Society of Health and Physical Educators | 1 | Important society for health and physical education professionals (“To advance professional practice and promote research related to health and physical education, physical activity, dance and sport”) |
| 18 | IPLA | International Physical Literacy Association | 1 | Specific network to promote physical literacy across the world (“Facilitate and support the adoption and promotion of physical literacy in communities worldwide”) |
| 19 | IPLC | The International Physical Literacy Conferences | 1 | Specific network to promote the exchange on physical literacy (“to bring together health, education, recreation and sport experts to advance the knowledge, application and implementation of physical literacy programming across the globe”) |
| 20 | NAHPL | National Academic of Health and Physical Literacy | 2 | Specific network in North America to promote health and physical literacy  (“advancing the dimensions of health and physical literacy through professional development, advocacy, mentoring, and applied research”). |

Note: The academic societies could nominate one or two representatives for the development process.
*Ultimately,the representative for ISPAH was unable to participate in the development process; however, the society has declared to continue promoting the dissemination.

*Electronic Supplementary Material 4.* Visualisation of the themes (bright) and overarching themes (dark) of the ‘advocacy’ meta-theme.

Note: The green lines highlight strongly compatible connections between (overarching) themes, while the red lines outline potential conflicts requiring discussion; this visualisation (i.e., the result of the pre-Delphi exercise) was presented at the beginning of the first group Delphi meeting.
PL = Physical Literacy; SDGs = Sustainable Development Goals.

*Electronic Supplementary Material 5.* Visualisation of the themes (bright) and overarching themes (dark) of the ‘practice’ meta-theme.

Note: This visualisation (i.e., the result of the pre-Delphi exercise) was presented at the beginning of the first group Delphi meeting.
PL = Physical Literacy

*Electronic Supplementary Material 6.* Visualisation of the themes (bright) and overarching themes (dark) of the ‘education’ meta-theme.

Note: This visualisation (i.e., the result of the pre-Delphi exercise) was presented at the beginning of the first group Delphi meeting.
PL = Physical Literacy.

*Electronic Supplementary Material 7.* Visualisation of the themes (bright) and overarching themes (dark) of the ‘assessment’ meta-theme.

Note: The green lines highlight strongly compatible connections between (overarching) themes, while the red lines outline potential conflicts requiring discussion; this visualisation (i.e., the result of the pre-Delphi exercise) was presented at the beginning of the first group Delphi meeting.
PL = Physical Literacy.

*Electronic Supplementary Material 8.* Visualisation of the themes (bright) and overarching themes (dark) of the ‘research’ meta-theme.

Note: The green lines highlight strongly compatible connections between (overarching) themes; this visualisation (i.e., the result of the pre-Delphi exercise) was presented at the beginning of the first group Delphi meeting.
PE = Physical Education; PL = Physical Literacy; SES = Socioeconomic status.

*Electronic Supplementary Material 9.* The timing of the meetings and their preparations.

|  | **Decisive preparatory meetings  (within the entire core team)** | **Pacific/American round** | **European/African/Asian round** |
| --- | --- | --- | --- |
| **Meeting 1** | 6^th^ November 2024, 5:30pm (EDT, GMT -4) 6^th^ November 2024, 9:30pm (GMT) ***7^th^ November 2024, 8:30am (AEDT, GMT +11)*** | ***12^th^ November 2024, 6pm (EDT, GMT -4)*** 12^th^ November 2024, 10pm (GMT) ***13^th^ November 2024, 9am (AEDT, GMT +11)*** | 14^th^ November 2024, 4am (EDT, GMT -4) ***14^th^ November 2024, 8am (GMT)*** 14^th^ November 2024, 7pm (AEDT, GMT +11) |
| **Meeting 2** | 29^th^ November 2024, 5pm (EDT, GMT -4) 29^th^ November 2024, 9pm (GMT) ***29^th^ November 2024, 8am (AEDT, GMT +11)*** | ***12^th^ December 2024, 6pm (EDT, GMT -4)*** 12^th^ December 2024, 10pm (GMT) ***13^th^ December 2024, 9am (AEDT, GMT +11)*** | 10^th^ December 2024, 4am (EDT, GMT -4) ***10^th^ December 2024, 8am (GMT)*** 10^th^ December 2024, 7pm (AEDT, GMT +11) |
| **Meeting 3** | 19^th^ January 2025, 9pm (EDT, GMT -4) 20^th^ January 2025, 12am (GMT) ***20^th^ January 2025, 11am (AEDT, GMT +11)***  22^nd^ January 2025, 10pm (EDT, GMT -4) 23^rd^ January 2025, 2am (GMT) ***23****^rd^* ***January 2025, 1pm (AEDT, GMT +11)*** | ***27^th^ January 2025, 6pm (EDT, GMT -4)*** 27^th^ January 2025, 10pm (GMT) ***28^th^ January 2025, 9am (AEDT, GMT +11)*** | 29^th^ January 2025, 4am (EDT, GMT -4) ***29^th^ January 2025, 8am (GMT)*** 29^th^ January 2025, 7pm (AEDT, GMT +11) |
| **Dissemination workshop** | Planned for June 2025 | Planned for July 2025 | Planned for July 2025 |

Note: The times are indicated for New York, USA (Eastern Daylight Time [EDT]; GMT -4); London, United Kingdom (Greenwich Mean Time [GMT]); and Melbourne, Australia (Australian Eastern Daylight Time [AEDT]; GMT +11). The times highlighted in bold are the times for which this round was specifically scheduled. Please consider that only the decisive preparatory meetings with the entire core team are listed, not with selected researchers.

*Electronic Supplementary Material 10.* Follow-up question specifying to which sustainable development goals (SDGs) the physical literacy concept connects.

| **Sustainable development goals (SDG)** | **Absolute votes** | **Relative votes** |
| --- | --- | --- |
| SDG-1: No Poverty | 0 | 0.0% |
| SDG-2: Zero Hunger | 0 | 0.0% |
| SDG-3: Good Health and Well-Being | 22 | 100.0% |
| SDG-4: Quality Education | 20 | 90.9% |
| SDG-5: Gender Equality | 13 | 59.1% |
| SDG-6: Clean Water and Sanitation | 1 | 4.5% |
| SDG-7: Affordable and Clean Energy | 2 | 9.1% |
| SDG-8: Decent Work and Economic Growth | 4 | 18.2% |
| SDG-9: Industry, Innovation, and Infrastructure | 1 | 4.5% |
| SDG-10: Reduced Inequalities | 13 | 59.1% |
| SDG-11: Sustainable Cities and Communities | 14 | 63.6% |
| SDG-12: Responsible Consumption and Production | 3 | 13.6% |
| SDG-13: Climate Action | 9 | 40.9% |
| SDG-14: Life Below Water | 1 | 4.5% |
| SDG-15: Life on Land | 4 | 18.2% |
| SDG-16: Peace, Justice, and Strong Institutions | 6 | 27.3% |
| SDG-17: Partnership for the Sustainable Development Goals | 4 | 18.2% |

Note: Nine of 31 participants expressed that they were lacking the specific expertise to select among the SDGs and, therefore, did not enter the voting; SDG-3, SDG-4, SDG-5, SDG-10, and SDG-11 were finally mentioned within the Global Physical Literacy (GloPL) Action Framework.
